# Collective voices, healthier outcomes: the union effect in American Healthcare

**DOI:** 10.1038/s44401-026-00078-z

**Published:** 2026-04-07

**Authors:** Kiran Abraham-Aggarwal, Yaminette Díaz-Linhart, Adam Seth Litwin, Ariel Avgar

**Affiliations:** 1 Department of Medicine, Weill Cornell Medicine, New York, NY USA; 2 School of Industrial and Labor Relations, Cornell University, Ithaca, NY USA; 3 Sloan School of Management, Massachusetts Institute of Technology, Cambridge, MA USA

**Keywords:** Health care, Social policy

## Abstract

Healthcare unionization is growing amid consolidation, burnout, and policy gridlock. Emerging evidence suggests unions improve healthcare delivery by enhancing worker conditions, staffing, and safety, which in turn support patient and public health outcomes. As physician organizing expands, unions play a vital role in advancing workplace standards, amplifying collective voice, and promoting a more equitable healthcare system.

Across industries, union petitions have doubled since 2021. Between 2023 and 2024, petitions rose by 27%^1^. Similarly, healthcare labor unionization has reached historic highs; 70,000 more healthcare workers unionized in 2024 than in 2023^2^. Catalyzed by consolidation, financialization of health systems, and the aftershocks of the COVID-19 pandemic, healthcare workers—from physicians to pharmacists—are organizing to gain greater voice over their work and working conditions^3^.

Further, labor organizing in healthcare is unfolding in a context increasingly inhospitable to constructive policymaking. Provider organizations, particularly those now under private equity or investor control, are disincentivized from implementing reforms that do not yield immediate financial returns^4^. In this environment, unions function less as a vehicle for system-wide efficiency and more as a practical lever for worker voice, staffing, and safety. While the major inefficiencies in U.S. healthcare reflect structural features such as high administrative costs relative to peer nations, unions can still help align day-to-day operations with frontline expertise and patient safety, complementing rather than substituting for broader policy reforms.

Of course, the very factors fueling union growth (consolidation, financialization, and workforce distress) complicate efforts to evaluate union effects independently of these underlying pressures^5^. In this sense, unionization is both a response to systemic dysfunction and a potential remedy for it^6^. Evidence across industries and historical periods suggests that, once established, unions often generate measurable improvements in working conditions, health outcomes, and equity; benefits that extend beyond the crises that gave rise to them^7,8^.

Given the shift toward unionization as a means of increasing worker voice and reshaping organizational priorities, we seek to analyze the question: *what do unions do in healthcare?* This Perspective reviews existing empirical evidence on the impact of unions in shaping working conditions, patient outcomes, and the broader public health.

## Healthcare: union strong?

The healthcare sector in the U.S. continues to suffer from poor patient outcomes and workforce health and safety issues, lagging behind similar advanced economies. Even with efforts to improve patient care quality and access, the U.S. ranks 55th out of 227 for infant mortality, 65th out of 186 for maternal mortality, 49th out of 227 in life expectancy at birth^9^. Workers in healthcare also experience their fair share of problems. Median earnings for healthcare workers for the entire healthcare and social assistance sector fall below the median earnings across all U.S. occupations^10^. Direct care workers, such as home health aides and personal care aides, also lack health insurance benefits, even with greater than average projected occupational growth^11,12^. Occupational safety concerns and injuries are on the rise, with healthcare workers experiencing an inordinate amount of stress from their work^13^. Many healthcare workers are experiencing higher rates of burnout and poor well-being^14,15^. Together, these pressures are felt across a heterogeneous workforce: registered nurses (RNs), allied health professionals, direct care workers in long-term care and home care, and physicians—each represented by different unions and bargaining structures. Their interests often align on staffing, safety, and scheduling, but can diverge around compensation models, scope of practice, and managerial autonomy.

In hospitals and health systems, RNs and many allied professionals typically bargain through nursing unions and multi-occupation healthcare unions, with priorities centered on safe staffing, injury prevention, and fatigue mitigation^16^. Direct care workers in long-term care and home care (home health aides, Certified Nursing Assistants, personal care aides) often organize through large, multi-industry unions, emphasizing wages, benefits, training, and anti-retaliation protections given historically low pay and high turnover^17,18^. Physicians, still a small minority of union members, organize mainly in trainee unions and a growing number of staff-physician units; their agendas skew toward clinical autonomy, workload intensity, and patient-care authority rather than wage bargaining^3,19,20^. Across groups, common mechanisms include enforceable staffing standards, safety protocols, limits on mandatory overtime, benefits coverage, and formal channels for voice^7^. Points of tension can emerge across these groups of workers when unit-specific gains create disparities across nonunion units or when productivity targets conflict with clinical judgment^3,21^.

Within this diverse union landscape, and despite overall declines in union density, healthcare unions have maintained a modest but durable presence^3,22^. More broadly, unions are well-documented to raise wages, expand access to benefits, strengthen occupational safety, and reduce inequality, mechanisms linked to better health and well-being for workers and communities^17,18,23–27^. In healthcare specifically, union activity is associated with safer staffing and injury reduction, greater Occupational Safety and Health Administration (OSHA) reporting compliance, lower turnover in nursing homes, adoption of patient-benefiting technologies, and improvements on nurse-sensitive quality indicators and risk-adjusted mortality^26,28–31^.

Against that backdrop, nationally representative evidence indicates that even at roughly 13% membership coverage of workers (2009–2021), unionization delivers measurable gains for U.S. healthcare workers: higher weekly earnings, more generous employer health-insurance contributions, and greater access to pensions and other retirement benefits, with especially pronounced effects for nurses and technicians^3^. Importantly, the study also found that unionization reduced racial pay disparities. While nonunionized White workers earned more than their racial minority peers, this gap was not observed among unionized workers^3^. Taken together, this literature suggests that incremental increases in unionization in U.S. healthcare may yield improvements in compensation, benefits, and staffing and safety culture, all of which plausibly support workforce stability and patient care quality, with effects varying by occupation (e.g., economic gains for nurses and technicians and autonomy for physicians).

Beyond wages and staffing, unions also influence how care is coordinated and delivered within organizations^32^. Research on high-performance healthcare systems highlights the importance of relational coordination, defined by shared goals, shared knowledge, and mutual respect across professional roles, in supporting effective, integrated care delivery^33^. In hospital settings, union-supported forums for employee voice and labor–management collaboration have been shown to facilitate care innovation, reduce turnover intentions, and improve organizational performance by strengthening coordination across frontline roles^34,35^. These mechanisms suggest that unions can contribute to care integration not only by improving staffing levels, but also by fostering the relational and organizational conditions necessary for patient-centered, team-based care^36^.

In addition to shaping care processes and coordination within healthcare organizations, labor unions have significantly improved working conditions for U.S. healthcare workers, particularly around staffing and safety. In states with enforceable nurse-to-patient ratios, such as California, mandated staffing standards are linked to lower burnout and fewer injuries—outcomes made possible through sustained union advocacy^37,38^. Unionized facilities also show stronger compliance with OSHA reporting, with nursing homes 31 percentage points more likely to submit data within two years of unionization^28^. Unions have likewise helped mitigate burnout and scheduling issues by lobbying to secure regulatory limits on mandatory overtime and ensuring rest periods, measures that directly address worker fatigue and moral distress^39,40^. These protections give healthcare workers more control over their time, promote mental health, and increase patient safety.

Strikes are a central and contested tool through which healthcare unions seek leverage to improve staffing, safety, and working conditions^41^. As temporary work stoppages, strikes raise legitimate concerns about patient safety and professional responsibility, and empirical evidence reflects this tension: in New York State, hospital strikes involving nurses were associated with a 19.4% increase of in-hospital mortality and a 6.5% rise in 30-day readmissions for patients admitted during strike periods^42^. In contrast, a study of physician strikes in the United Kingdom found no overall impact on emergency readmissions or inpatient mortality, although differential effects were observed for Black patients^43^. To address these concerns, unions often limit strikes to short, fixed durations, provide advance notice, and maintain emergency coverage, while reframing collective action as consistent with professional ethics when undertaken to protect safe care conditions^44–46^. As Krachler et al. show, nurse unions explicitly link professionalism to patient advocacy, arguing that withdrawing labor in the face of unsafe staffing can be a duty of care rather than a violation of it^47^.

Beyond strikes, studies link unionization to better care quality. Dube et al. found unionized hospitals outperformed nonunion ones on 12 of 13 nurse-sensitive outcomes, including infections and failure-to-rescue^48^. Likewise, Ash and Seago reported lower heart attack mortality in hospitals with long-standing nurse unions, underscoring how stable collective voice improves care quality^49^.

Existing studies, while limited in number and in scope, point to pathways through which unions can benefit both workers and patients, chiefly by improving working conditions that, in turn support patient care, quality, and safety. In many settings, unions prioritize patient safety, staffing adequacy, and long-term workforce health, even when these goals do not directly align with financial incentives. They may also prompt hospital investments in socially beneficial innovations, particularly in under-resourced institutions where such investments might otherwise be delayed^30^. Though some literature has found that the net impact of unions depends on institutions and management-union relations, broader findings have identified that unions can help steer organizations toward welfare-enhancing decisions in healthcare settings^50^. As Ash et al. note: unions benefit patients in the same vein as workers because “health workers’ working conditions are patients’ care conditions, their interests often align on issues of staffing, scheduling, and process.” (p. 393)^51^ While the preceding section highlights the state of unionization and its impacts within U.S. healthcare, comparing these dynamics across other countries offers critical perspective on both potential and limitations of organized labor in shaping healthcare outcomes.

## International comparisons of healthcare unionization

Evidence from outside the United States shows that unions improved working conditions, increased job satisfaction for workers, and contributed to gains in quality of care for patients, particularly in regions with supportive labor laws and healthcare funding^52–59^. Notably, healthcare unionization is often much stronger in other countries than in the United States. Across OECD member countries, union density varies widely: while the United States has a relatively low overall union density of about 10%, several European countries, including Sweden, Denmark, Norway, and Iceland, report union membership rates above 60% with up to over 90% of workers belonging to unions^60–62^. Collective bargaining coverage is also substantially higher in many European systems due to multi-employer and sectoral agreements, compared to less than 20% coverage in the United States. In these contexts, unions have a broader platform to influence workforce conditions (e.g., national wage agreements) and even participate in governance through co-determination rights (as in Germany, where worker representatives have formal decision-making roles)^63^. These institutional supports give healthcare unions greater leverage to secure safer staffing, higher pay, and comprehensive policy reforms, resulting in even more pronounced benefits for workers and patients abroad. For example, one study in the US found that outsourcing hospital cleaning staff (often eliminating unionized jobs) led to higher infection rates, underscoring how stable, union-supported employment can protect patient safety^32,47,63,64^. Similarly, greater frontline staff engagement in hospitals has been linked to lower error rates and improved care perceptions, illustrating the positive impact of worker voice on care quality^34^. At the same time, union effectiveness varies by broader social, economic, and political contexts in which unions operate^65^. In the literature, union influence extends to patient outcomes, worker outcomes, and healthcare policy outcomes, underscoring that the translation of worker voice into care quality depends on both institutional design and organizing intensity.

Across the scope of the literature, unions serve as a vital mechanism for promoting workplace safety and ensuring adherence to occupational standards, with downstream effects on care quality^66–68^. In the United Kingdom, healthcare unions played a central role during the COVID-19 crisis by securing personal protective equipment and advancing infection-control measures for frontline workers. At the same time, evidence from the National Health Service shows that market-oriented reforms such as the Private Finance Initiative tested these protections and required extensive bargaining over employment conditions^56,69^. Bach and Givan demonstrate that unions were central to renegotiating work rules, staffing arrangements, and employment protections during these restructurings, helping to preserve workforce stability and care standards amid organizational change^69^. By contrast, in parts of Southern Europe, prolonged fiscal austerity and chronic underfunding constrained unions’ capacity to uphold safety standards consistently, resulting in uneven protections across healthcare settings^53^. Together, these cases show how structural and financial conditions shape unions’ capacity to secure safer workplaces. They also determine whether unions can sustain the long-term practices needed for a resilient healthcare workforce^44^.

Worker well-being and compensation exhibit similar patterns. Unions play a critical role in mitigating stress among healthcare workers by advocating for better working conditions. Many workers describe membership as a source of protection and solidarity^17,70^. However, some studies in the United States have found only partial gains, particularly in training environments where burnout may not improve despite better material benefits^71^. Globally, unions have advanced health policies benefiting workers, patients, and communities^8,72–75^. In Canada, higher unionization rates over 20 years were linked to lower total, preventable, and treatable mortality^76^. From infection prevention standards to occupational safety programs, organized labor has driven systemic reforms that strengthen care quality and equity, underscoring the value of embedding collective voice within national healthcare frameworks. Indeed, collaborative labor-management approaches in healthcare can yield mutual gains: union–management partnerships have enhanced employee voice and problem-solving in hospitals, and studies show that formal union participation can complement other employee-involvement initiatives to improve organizational performance^64^. This aligns with the broader principle that strong relationships among healthcare staff, often bolstered by unions, are key to high-performance, resilient care systems^33^.

## Impact of unions on health outcomes

Considering the role of unions in enhancing working conditions, promoting worker well-being and increase patient care quality, unionization may also impact broader health outcomes. Two historical communicable disease cases (one in the U.S. and the other in Australia) point to creative advocacy strategies and partnerships that led to successful infection prevention guidelines for healthcare workers, patients and even public workers at risk for infection (i.e., police, sanitation workers etc.) by requiring access to vaccines through employing organizations and^73–75^ Although these cases are quite limited in their generalizability, they may serve as examples for future research focused on capturing union involvement in advocacy and policymaking in the healthcare industry.

More broadly, recent research points to the impact of unionization on health outcomes. A recent review of union effects on health of workers across the U.S., Europe and Canada, found evidence that unions likely improve the health of individuals broadly through access to health insurance, increased wages, improving working conditions, increased safety reporting, and increased access to benefits, such as pensions, overtime, sick leave, or on the job-training^7^. Relatedly, an analysis of almost 2-million workers across the U.S. and Europe found that union membership was positively associated with job satisfaction, life satisfaction, and negatively associated with other markers of well-being, including stress and depression^77^. Extending well-being markers beyond satisfaction, a mixed-methods study found that union instrumentality reduced workers’ emotional exhaustion through union involvement in management practices via collective bargaining provisions, consultation, and grievances to ensure management followed-through with implementation of their provisions^78^. Finally, a novel study focused on the physical health of older adults, found positive impacts of cumulative unionization (i.e., accumulated union membership across one’s career) with health outcomes, whereby respondents who were union members throughout their entire careers having better health outcomes (self-rated health, functional limitations and chronic health conditions)^79^. As this area of research continues to grow, current research points to the potential that union membership has to promote the health and well-being of individuals across a variety of industries.

## Trends in physician unionization

While much of the existing research has focused on nurses and allied health professionals, the question of unionization is increasingly relevant for physicians^80^. Physicians, once rarely unionized, are now increasingly joining the labor movement. For example, approximately 8% of U.S. physicians (roughly 72,000) are union members today^81^. In the past, only 0–6 physician union petitions were filed annually, but this jumped to 21 new physician union drives in 2023 and another 12 in the first five months of 2024^81^. These recent drives alone, if successful, would unionize about 3523 physicians—nearly equal to the total number of doctors who sought union representation in the prior 22 years^81^. What’s also important is that the drivers of physician unionization appear to be non-financial: surveys of recent physician organizing efforts show that working conditions (cited in 85% of cases), lack of voice in management (81%), and patient care concerns (54%) far outweigh pay (4%) as motivations^81^.

Even the American Medical Association (AMA) has taken note, formally supporting physicians’ collective bargaining rights under the NLRA^80^. According to the AMA’s 2023 ARC Issue Brief, employed physicians enjoy protections under the NLRA and may form or join bargaining units, even without being part of a traditional union. Despite this, physician union density remains low: just 7.2% of practicing physicians were union members in 2019^82^. Still, this number has grown significantly from prior decades, especially among younger or hospital-employed physicians^80^. The Union of American Physicians and Dentists and the Committee of Interns and Residents (which is AMA supported), now represents about 20% of all U.S. physician trainees (32,000)^83^. Moreover, a 2025 national survey found that among resident physicians not currently unionized, 63% would vote for union representation, indicating substantial pent-up demand for collective voice^84^.

As the AMA highlights, there are several factors fueling physician unionization: burnout, lack of autonomy, and administrative overload. Studies cited by the AMA have shown that physicians today spend twice as much time on documentation as they do on direct patient care, and younger physicians are increasingly attracted to collective bargaining as a way to restore work-life balance and regain control over practice conditions^85–87^. As physicians shift toward unionization, future research should highlight the specific impacts of unionization on physicians, their working conditions and impacts on patient care.

## Union partnerships

While union effectiveness may be contingent on the shifting economic and labor landscape, unions *do* improve working conditions, which in turn can improve patient safety and care. Through increasing wages and access to benefits, unions improve health of members and nonunion individuals. Beyond the workplace, union advocacy has shaped health policy with spillovers for patients and communities. While strikes often draw attention, the more routine and consequential work occurs through collaboration, unit-based teams, joint training funds, and co-developed safety standards. Given this track record, a practical next step is to expand creative union–management partnerships that align worker voice with care improvement.

Policymakers, public health practitioners, and healthcare leaders should consider creative partnerships with labor unions that find common ground and deliver mutual gains for patients, workers and communities. Table 1 showcases examples of existing union partnerships that focus on gains for workers, and the broader public. Healthcare systems, like Kaiser Permanente and UMass Memorial have successful long-standing labor-management programs. Multistakeholder programs with unions improve access to training, professional development and career advancement for healthcare workers targeting care and job quality. Union collaborations have also paved the way for legislation that has had profound impacts on the public, including regulations and legislation for smoke-free airlines, as well as the minimum nurse staffing ratio mandate in California, demonstrating significant reductions in patient mortality and improvements in nurse well-being. Much of this work remains empirically underexplored—underestimating union effectiveness.

**Table 1:** Example union strategies and partnerships

Strategies	Examples
Unit based teams composed of union representative, employees, managers, & providers focused on patient care and work process improvements	Kaiser Permanente SHARE-UMass Memorial
Multistakeholder industry partnerships developing and implementing training programs that build career pathways and skills for frontline healthcare workers	Healthcare Career Advancement Program Massachusetts 1199SEIU Training and Upgrading Fund
Collaborative advocacy to improve access to health insurance and improve workplace health and safety	California Nurses Association and minimum staffing ratio legislative mandates (A.B. 394) Association of Flight Attendants and Federal Aviation Administration regulations for smoke-free airlines

For example, Kaiser Permanente’s Labor Management Partnership has established “unit-based teams” of frontline employees and managers, a model credited with improving workers’ willingness to speak up about safety and with enhancing workplace health culture^88^. At the University of Massachusetts Memorial Health, a similar partnership with the SHARE union has created over 50 labor-management unit-based teams since 2017, yielding measurable improvements in caregiving processes, staff morale, organizational culture, and even the Hospital’s bottom line^88^. Meanwhile, 1199SEIU’s Training and Employment Funds (TEF), one of the nation’s largest healthcare industry partnerships, invest in workforce development and have helped thousands of workers upgrade their skills and advance their careers in healthcare. Demonstrating the impact of such investments, a study by Sterling et al. evaluated a TEF-supported program that trained home health aides to recognize signs of heart failure, enhancing both care quality and worker confidence^89–91^. The opportunities and services provided by unions can help translate to safer, higher-quality care and more sustainable workforce outcomes by boosting employee engagement, reducing turnover, and fostering a more skilled workforce.

Even so, union partnerships remain empirically underexplored. Eaton et al.^35^ described the history of labor management cooperation in the United States and found that these partnerships are rare, hard to sustain, and their collaborative work not often designated as “partnerships.” An early case study on successful union-management collaboration emphasized employee involvement and mutual accommodation of all stakeholder interest as important factors in partnerships; elements of success included: visible support from top management and the labor union, training resources, and empowered employees^92,93^. Finally, Malinowski et al.^94^ described several examples of successful union labor management partnerships in access to screening, detection and treatment of hypertension, or asthma-related surveillance programs and workplace asthma interventions^93^. Additional research is needed to understand detailed elements of successful partnerships, as well as rigorous evaluation of outcomes resulting from these collaborations.

## Study limitations

This Perspective draws together a heterogeneous literature that is still emerging, often observational, and concentrated in certain occupations (notably nurses and direct-care workers) and contexts (e.g., California). These features naturally temper causal claims and generalizability, but they also highlight where evidence is strongest and where replication is most feasible. Estimates may be shaped by selection and unmeasured factors (management quality, financing, market structure), and several outcomes, especially strike effects, are sensitive to strike type, duration, services maintained, and hospital mitigation strategies. Some indicators (e.g., OSHA reporting, nurse-sensitive measures) serve as proxies and can be influenced by documentation practices. Evidence on physician unionization is nascent but rapidly expanding. International findings are used for context; system differences limit one-to-one comparisons but offer useful new directions for U.S. policy. Overall, these constraints point to clear opportunities: more longitudinal and quasi-experimental studies across diverse occupations and jurisdictions, routine reporting of neutral/null results, and deeper tests of mechanisms linking worker voice to patient and public health outcomes.

## A window of opportunity?

In healthcare systems around the world, empowering worker voice through unions or formal representation has improved working conditions and the quality of care. Across diverse contexts, studies highlight staffing, safety, and equity gains when health workers have collective bargaining power. Conversely, excluding labor from key decisions can undermine safety and care quality. As healthcare systems confront increasingly complex challenges, strengthening collective worker voice represents a timely opportunity to improve outcomes for both caregivers and patients.

## Data Availability

No datasets were generated or analyzed during the current study.

## Generative artificial intelligence statement

This work was created entirely by the authors and in no way by artificial intelligence (AI). Please do not input this piece into ChatGPT or any other AI service.
